# The genome sequence of the light-bulb sea squirt, *Clavelina lepadiformis *(Müller, 1776)

**DOI:** 10.12688/wellcomeopenres.20417.1

**Published:** 2023-11-23

**Authors:** John Bishop, Christine Wood, Rob Mrowicki, Joanna Harley, Marine Biological Association Genome Acquisition Lab, Darwin Tree of Life Barcoding collective, Wellcome Sanger Institute Tree of Life Management, Samples and Laboratory team, Wellcome Sanger Institute Scientific Operations: Sequencing Operations, Wellcome Sanger Institute Tree of Life Core Informatics team, Tree of Life Core Informatics collective, Darwin Tree of Life Consortium

**Affiliations:** 1 The Marine Biological Association, Plymouth, England, UK

**Keywords:** Clavelina lepadiformis, light-bulb sea squirt, genome sequence, chromosomal, Aplousobranchia

## Abstract

We present a genome assembly from an individual *Clavelina lepadiformis* (the light-bulb sea squirt; Chordata; Ascidiacea; Aplousobranchia; Clavelinidae). The genome sequence is 210.1 megabases in span. Most of the assembly is scaffolded into 9 chromosomal pseudomolecules. The mitochondrial genome has also been assembled and is 14.48 kilobases in length.

## Species taxonomy

Eukaryota; Metazoa; Eumetazoa; Bilateria; Deuterostomia; Chordata; Tunicata; Ascidiacea; Aplousobranchia; Clavelinidae; *Clavelina*; *Clavelina lepadiformis* (Müller, 1776) (NCBI:txid159417).

## Background

*Clavelina lepadiformis* is a colonial (budding) ascidian (sea squirt) of the Order Aplousobranchia. The colony is formed by the repeated budding of zooids (‘individual’ sea squirt bodies) starting with the founding zooid formed by the metamorphosis of a swimming larva. The zooids of a colony remain linked basally; they are up to 45 mm tall, elongate in shape with the inhalant and exhalant siphons at the upper end, and are largely transparent, although the branchial basket and siphon rims are marked with white, yellow or pink lines and circles. This pigmentation accounts for the common name ‘Light-bulb sea squirt’, referring to the fancied resemblance of colonies to clusters of incandescent light bulbs with glowing filaments. As is typical of colonial ascidians, embryos are brooded until released as larvae.

*C. lepadiformis* grows on solid surfaces from the low shore down to c. 100 m, and is frequent in harbours. It ranges from southern Scandinavia to the Mediterranean, and is present as an introduced species in the Azores, South Africa, South Korea, Japan and on the US east coast ( Fofonoff *et al.*, 2018; Nishikawa & Namikawa, 2018). Molecular (COI sequence) evidence identified two distinct clades in the western Mediterranean, one in natural rocky habitats on the open coast and one in harbours and marinas ( Turon *et al.*, 2003); the form in harbours and marinas is very closely related to the clade found in both habitats in the European Atlantic, and seems to represent a recent anthropogenic introduction from the Atlantic. The clades could not be distinguished morphologically. However, de Caralt *et al.* (2002) found clear differences in the phenology of harbour and open rocky littoral populations of *C. lepadiformis* in the western Mediterranean. The harbour population brooded larvae from November to June with several gonadal cycles over that time. In contrast, the open-coast colonies brooded larvae for only 2 to 3 months in winter-spring, with a single gonadal cycle per year. The zooids of open coast colonies regressed over the summer, such summer inactivity (aestivation) being a pattern seen in a range of shallow-water marine invertebrates in the Mediterranean.

The zooids of *C. lepadiformis* appear particularly clean and transparent, lacking fouling by other sessile species. This may in large part reflect chemical defence against colonisation of their surface. Crude aqueous extracts of *C. intestinalis* tissue showed strong toxicity to a range of invertebrate larvae, greater than the effect of extracts from three other tunicate species ( Teo & Ryland, 1995). *C. intestinalis* produces a range of cytotoxic alkaloids: lepadins ( Casertano *et al.*, 2022; Kubanek *et al.*, 1995; Steffan, 1991); lepadiformine 1 ( Biard *et al.*, 1994); and villatamines ( Kubanek *et al.*, 1995). Several of these substances were also found in the flatworm *Prostheceraeus villatus*, which feeds on *C. lepadiformis* ( Kubanek *et al.*, 1995).

## Genome sequence report

**Figure 1.**
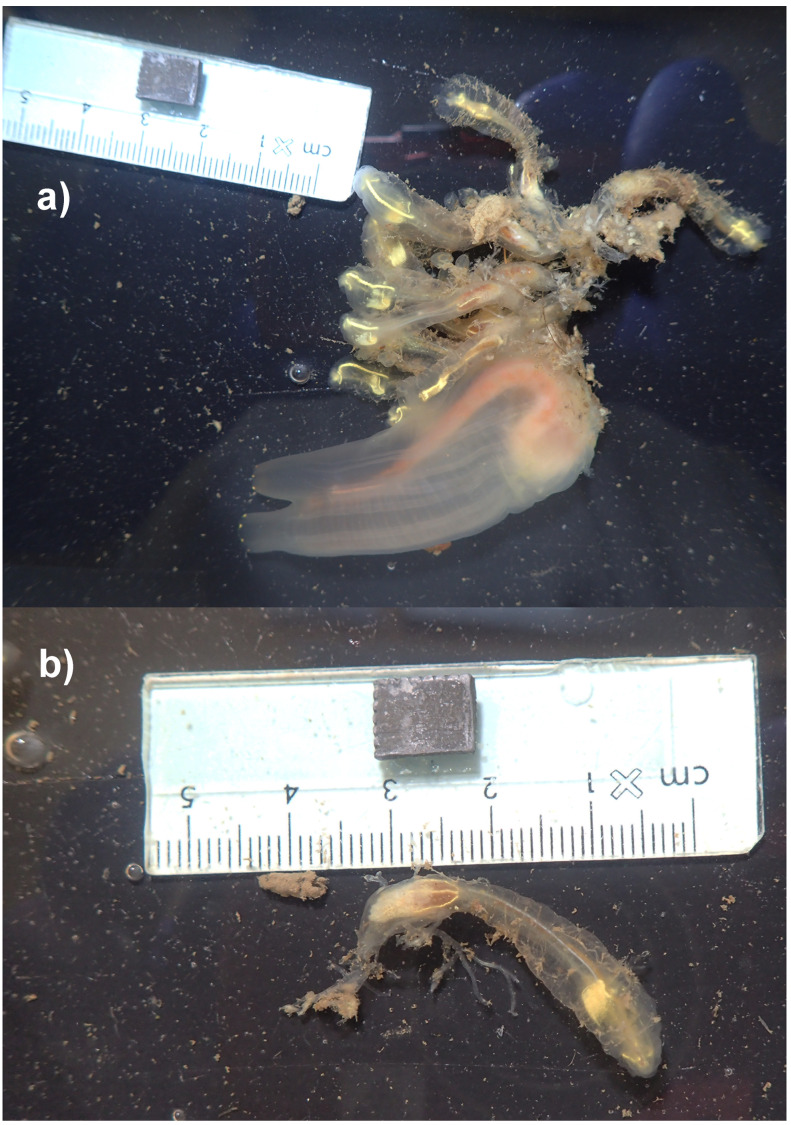
Photographs of the *Clavelina lepadiformis* (kaClaLepa1) specimen used for genome sequencing: **a**) Attached to specimen of *Ciona intestinalis*), **b**) Separated.

The genome was sequenced from *Clavelina lepadiformis* ( Figure 1) collected from Queen Anne's Battery Marina visitors’ pontoon, Plymouth, UK (50.36, –4.13). A total of 117-fold coverage in Pacific Biosciences single-molecule HiFi long reads was generated. Primary assembly contigs were scaffolded with chromosome conformation Hi-C data. Manual assembly curation corrected 60 missing joins or mis-joins and removed 9 haplotypic duplications, reducing the assembly length by 5.78% and the scaffold number by 25.9%, and increasing the scaffold N50 by 35.45%.

**Figure 2.**
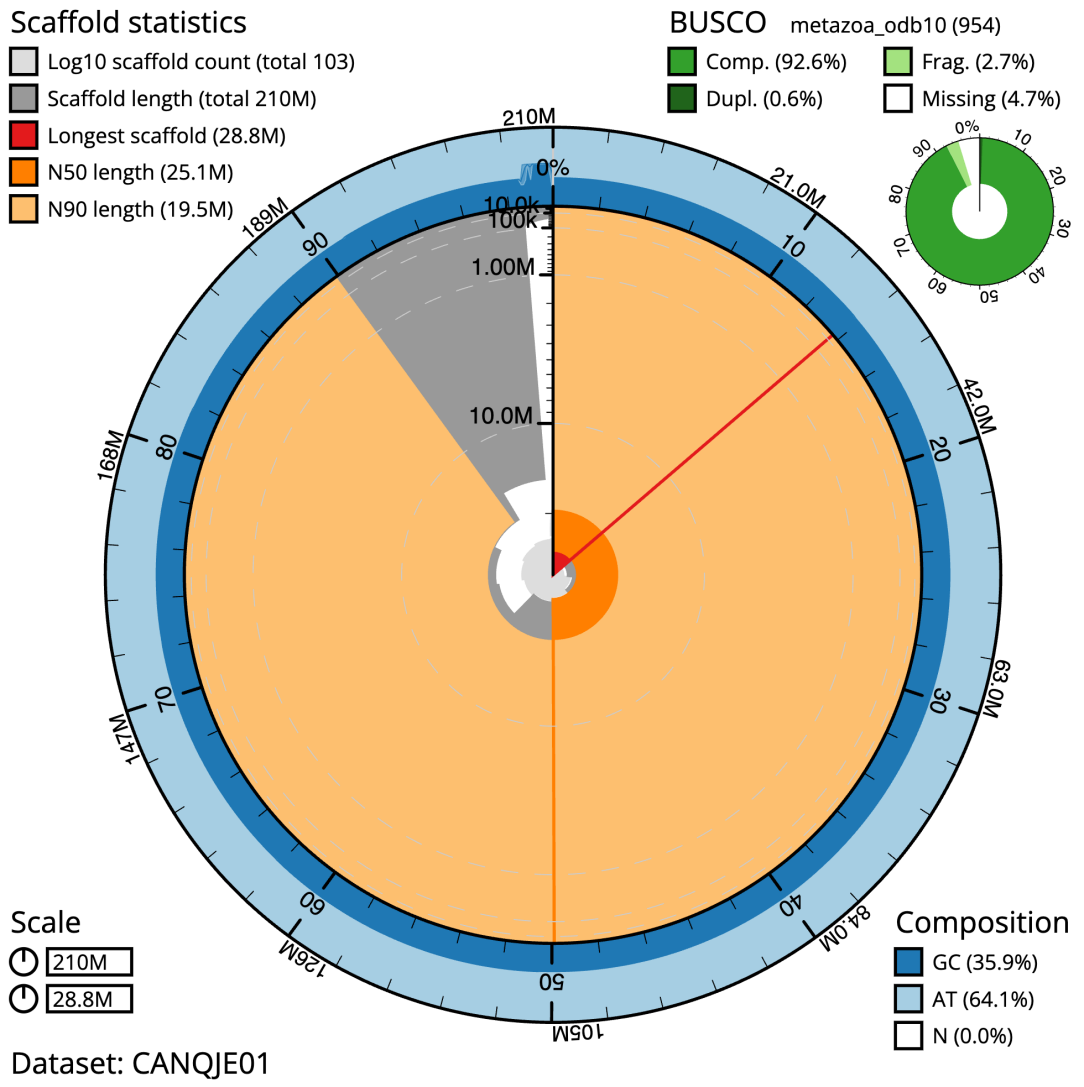
Genome assembly of *Clavelina lepadiformis*, kaClaLepa1.1: metrics. The BlobToolKit Snailplot shows N50 metrics and BUSCO gene completeness. The main plot is divided into 1,000 size-ordered bins around the circumference with each bin representing 0.1% of the 210,089,204 bp assembly. The distribution of scaffold lengths is shown in dark grey with the plot radius scaled to the longest scaffold present in the assembly (28,789,166 bp, shown in red). Orange and pale-orange arcs show the N50 and N90 scaffold lengths (25,070,715 and 19,509,797 bp), respectively. The pale grey spiral shows the cumulative scaffold count on a log scale with white scale lines showing successive orders of magnitude. The blue and pale-blue area around the outside of the plot shows the distribution of GC, AT and N percentages in the same bins as the inner plot. A summary of complete, fragmented, duplicated and missing BUSCO genes in the metazoa_odb10 set is shown in the top right. An interactive version of this figure is available at https://blobtoolkit.genomehubs.org/view/Clavelina%20lepadiformis/dataset/CANQJE01/snail.

**Figure 3.**
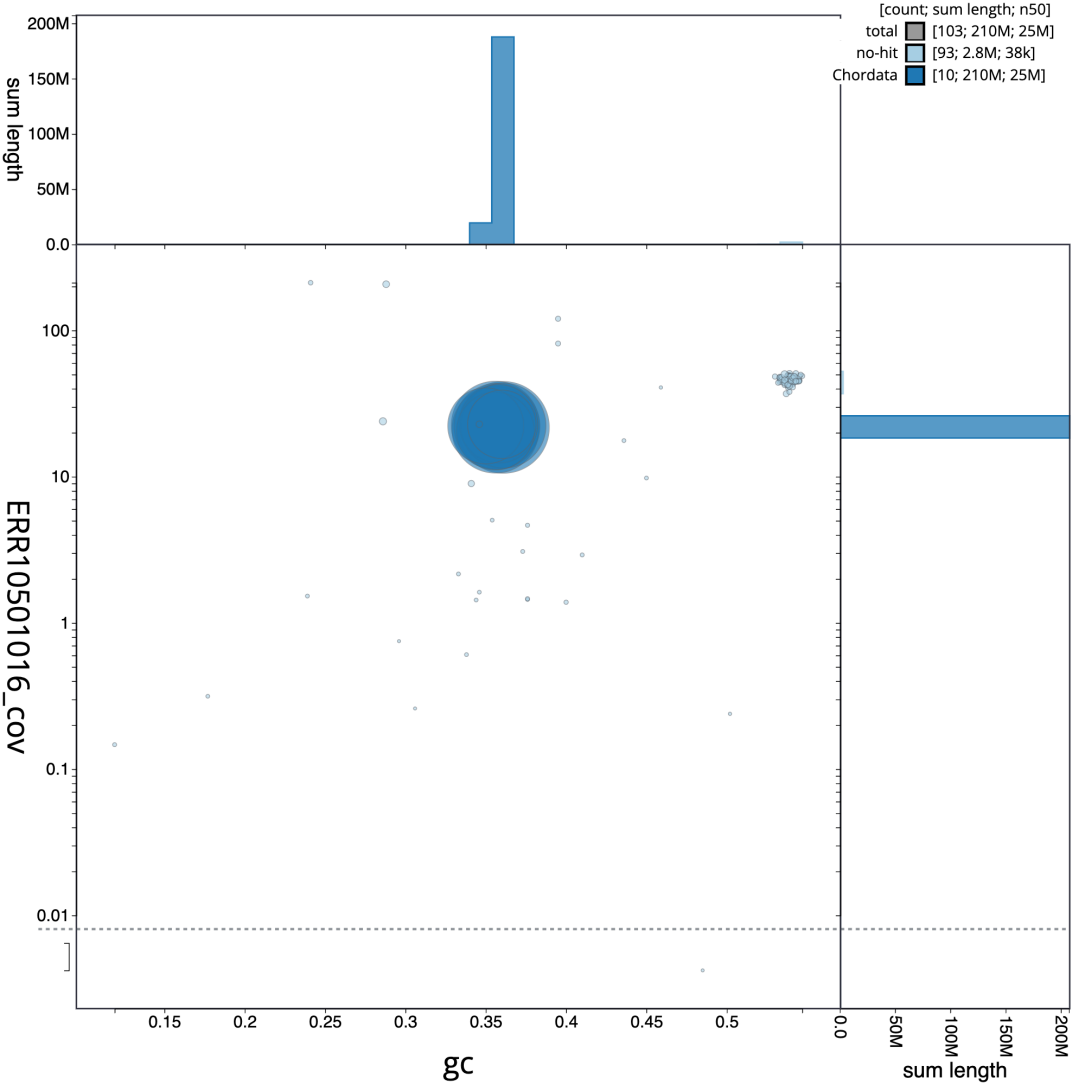
Genome assembly of *Clavelina lepadiformis*, kaClaLepa1.1: BlobToolKit GC-coverage plot. Scaffolds are coloured by phylum. Circles are sized in proportion to scaffold length. Histograms show the distribution of scaffold length sum along each axis. An interactive version of this figure is available at https://blobtoolkit.genomehubs.org/view/Clavelina%20lepadiformis/dataset/CANQJE01/blob.

**Figure 4.**
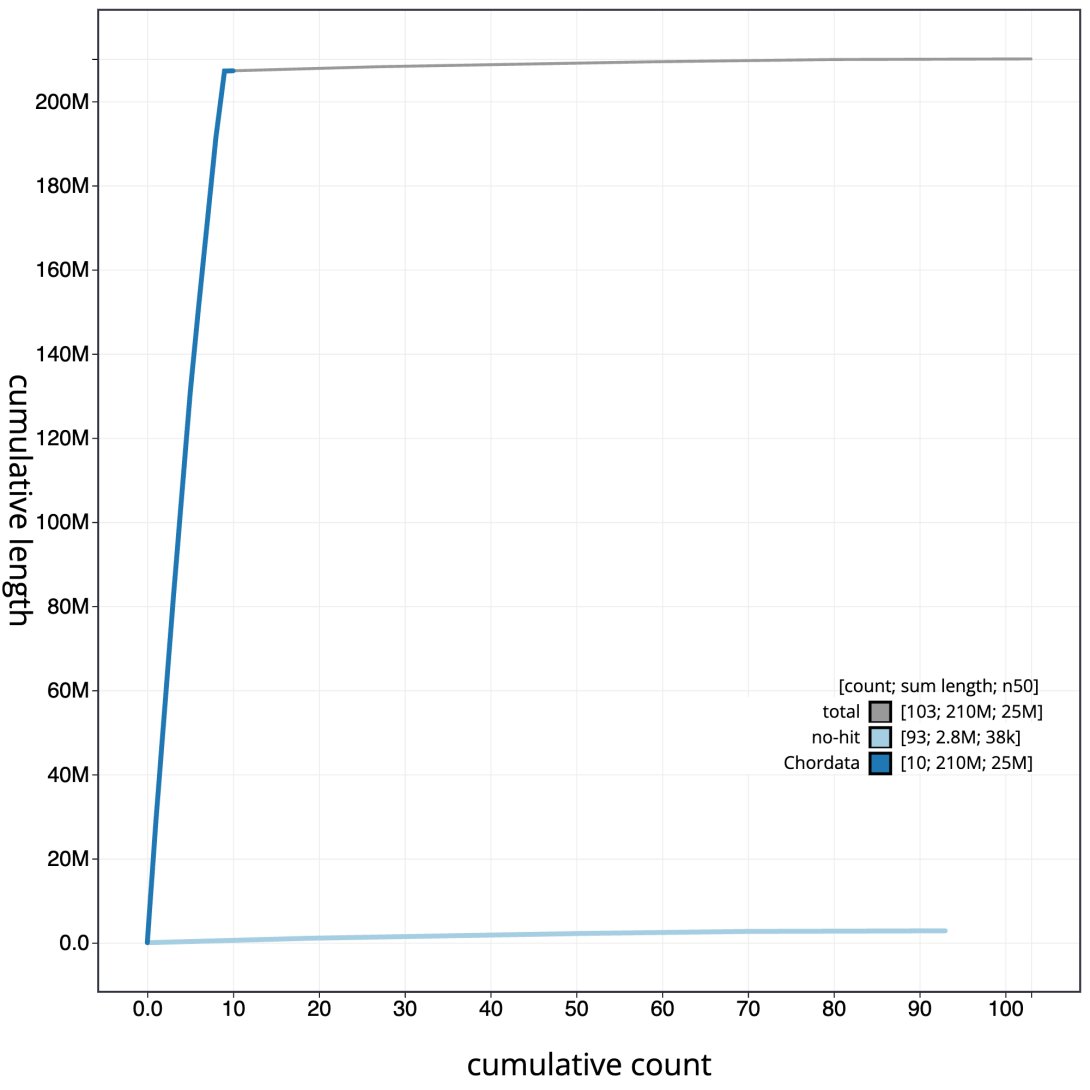
Genome assembly of *Clavelina lepadiformis*, kaClaLepa1.1: BlobToolKit cumulative sequence plot. The grey line shows cumulative length for all scaffolds. Coloured lines show cumulative lengths of scaffolds assigned to each phylum using the buscogenes taxrule. An interactive version of this figure is available at https://blobtoolkit.genomehubs.org/view/Clavelina%20lepadiformis/dataset/CANQJE01/cumulative.

**Figure 5.**
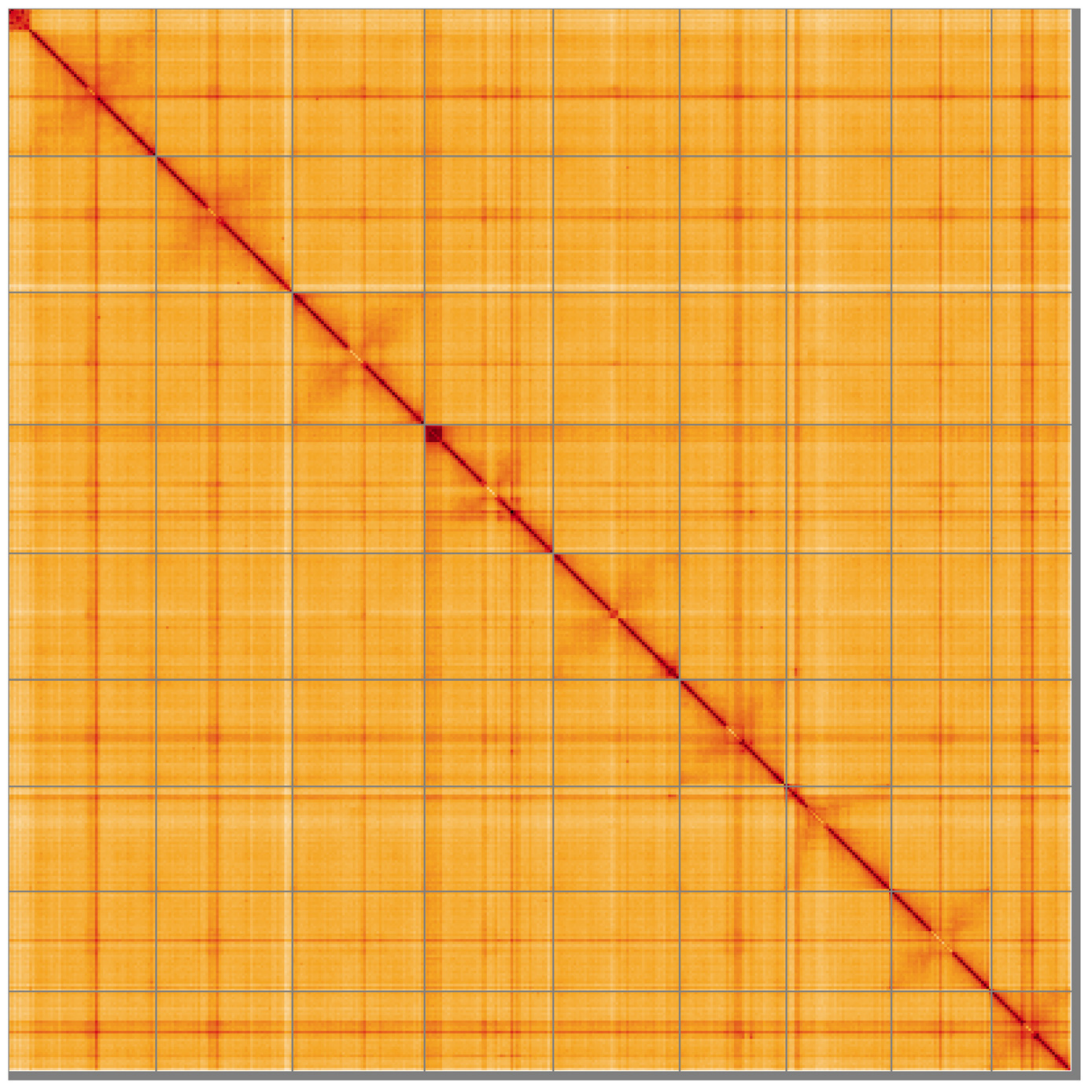
Genome assembly of *Clavelina lepadiformis*, kaClaLepa1.1: Hi-C contact map of the kaClaLepa1.1 assembly, visualised using HiGlass. Chromosomes are shown in order of size from left to right and top to bottom. An interactive version of this figure may be viewed at https://genome-note-higlass.tol.sanger.ac.uk/l/?d=X_VH7rrISNydJ-6yEBmyFw.

The final assembly has a total length of 210.1 Mb in 102 sequence scaffolds with a scaffold N50 of 25.1 Mb ( Table 1). The snailplot in Figure 2 provides a summary of the assembly statistics, while the distribution of assembly scaffolds on GC proportion and coverage is shown in Figure 3. The cumulative assembly plot in Figure 4 shows curves for subsets of scaffolds assigned to different phyla. Most (98.62%) of the assembly sequence was assigned to 9 chromosomal-level scaffolds. Karyotyping information was used from Colombera (1971). Chromosome-scale scaffolds confirmed by the Hi-C data are named in order of size ( Figure 5; Table 2). While not fully phased, the assembly deposited is of one haplotype. Contigs corresponding to the second haplotype have also been deposited. The mitochondrial genome was also assembled and can be found as a contig within the multifasta file of the genome submission.

**Table 1.** Genome data for *Clavelina lepadiformis*, kaClaLepa1.1.

Project accession data		
Assembly identifier	kaClaLepa1.1	
Assembly release date	2022-12-19	
Species	*Clavelina lepadiformis*	
Specimen	kaClaLepa1	
NCBI taxonomy ID	159417	
BioProject	PRJEB57668	
BioSample ID	SAMEA7536527	
Isolate information	kaClaLepa1 (DNA sequencing) kaClaLepa3 (Hi-C data) kaClaLepa9 (RNA sequencing)	
Assembly metrics *		*Benchmark*
Consensus quality (QV)	67.4	*≥ 50*
*k*-mer completeness	100%	*≥ 95%*
BUSCO **	C:92.6%[S:91.9%,D:0.6%], F:2.7%,M:4.7%,n:954	*C ≥ 95%*
Percentage of assembly mapped to chromosomes	98.62%	*≥ 95%*
Sex chromosomes	-	*localised homologous pairs*
Organelles	Mitochondrial genome assembled.	*complete single alleles*
Raw data accessions		
PacificBiosciences SEQUEL II	ERR10499356	
Hi-C Illumina	ERR10501018	
PolyA RNA-Seq Illumina	ERR10908613	
Genome assembly		
Assembly accession	GCA_947623445.1	
*Accession of alternate haplotype*	GCA_947623165.1	
Span (Mb)	210.1	
Number of contigs	161	
Contig N50 length (Mb)	8.3	
Number of scaffolds	102	
Scaffold N50 length (Mb)	25.1	
Longest scaffold (Mb)	28.8	

* Assembly metric benchmarks are adapted from column VGP-2020 of “Table 1: Proposed standards and metrics for defining genome assembly quality” from ( Rhie *et al.*, 2021). ** BUSCO scores based on the metazoa_odb10 BUSCO set using v5.3.2. C = complete [S = single copy, D = duplicated], F = fragmented, M = missing, n = number of orthologues in comparison. A full set of BUSCO scores is available at https://blobtoolkit.genomehubs.org/view/Clavelina%20lepadiformis/dataset/CANQJE01/busco.

**Table 2.** Chromosomal pseudomolecules in the genome assembly of *Clavelina lepadiformis*, kaClaLepa1.

INSDC accession	Chromosome	Length (Mb)	GC%
OX392439.1	1	28.79	36.0
OX392440.1	2	26.54	35.5
OX392441.1	3	25.74	35.5
OX392442.1	4	25.07	35.5
OX392443.1	5	24.62	36.0
OX392444.1	6	20.77	36.0
OX392445.1	7	20.45	35.5
OX392446.1	8	19.51	35.0
OX392447.1	9	15.73	36.0
OX392448.1	MT	0.01	24.0

The estimated Quality Value (QV) of the final assembly is 67.4 with *k*-mer completeness of 100%, and the assembly has a BUSCO v5.3.2 completeness of 92.6% (single = 91.9%, duplicated = 0.6%), using the metazoa_odb10 reference set ( *n* = 954).

Metadata for specimens, barcode results, spectra estimates, sequencing runs, contaminants and pre-curation assembly statistics are given at https://links.tol.sanger.ac.uk/species/159417.

## Methods

### Sample acquisition and nucleic acid extraction

Specimens of *Clavelina lepadiformis* were collected by hand from submerged rope from the Queen Anne's Battery Marina visitors’ pontoon, Plymouth, Devon, UK (latitude 50.36, longitude –4.13) on 2020-07-13, and were subsequently preserved in liquid nitrogen. The specimen was collected and identified by Christine Wood, John Bishop, Rob Mrowicki and Joanna Harley (Marine Biological Association). One specimen was used for DNA sequencing (specimen ID MBA-200713-001A, ToLID kaClaLepa1), another for Hi-C data (specimen ID MBA-200713-001C, ToLID kaClaLepa3) and another for RNA sequencing (specimen ID MBA-200713-001J, ToLID kaClaLepa9).

High molecular weight (HMW) DNA was extracted at the Tree of Life laboratory, Wellcome Sanger Institute (WSI), following a sequence of core procedures: sample preparation; sample homogenisation; HMW DNA extraction; DNA fragmentation; and DNA clean-up. The kaClaLepa1 sample was weighed and dissected on dry ice (as per the protocol https://dx.doi.org/10.17504/protocols.io.x54v9prmqg3e/v1). The sample was homogenised using a Nippi Powermasher fitted with a BioMasher pestle, following the protocol at https://dx.doi.org/10.17504/protocols.io.5qpvo3r19v4o/v1. DNA was extracted by means of the HMW DNA Extraction: Automated MagAttract protocol ( https://dx.doi.org/10.17504/protocols.io.kxygx3y4dg8j/v1). HMW DNA was sheared into an average fragment size of 12–20 kb in a Megaruptor 3 system with speed setting 30, following the HMW DNA Fragmentation: Diagenode Megaruptor®3 for PacBio HiFi protocol ( https://dx.doi.org/10.17504/protocols.io.8epv5x2zjg1b/v1). Sheared DNA was purified using solid-phase reversible immobilisation (SPRI) (protocol at https://dx.doi.org/10.17504/protocols.io.kxygx3y1dg8j/v1). In brief, the method employs a 1.8X ratio of AMPure PB beads to sample to eliminate shorter fragments and concentrate the DNA. The concentration of the sheared and purified DNA was assessed using a Nanodrop spectrophotometer and Qubit Fluorometer and Qubit dsDNA High Sensitivity Assay kit. Fragment size distribution was evaluated by running the sample on the FemtoPulse system.

RNA was extracted from kaClaLepa9 using the Automated MagMax™ *mir*Vana protocol ( https://dx.doi.org/10.17504/protocols.io.6qpvr36n3vmk/v1). The RNA concentration was assessed using a Nanodrop spectrophotometer and Qubit Fluorometer using the Qubit RNA Broad-Range (BR) Assay kit. Analysis of the integrity of the RNA was done using the Agilent RNA 6000 Pico Kit and Eukaryotic Total RNA assay.

All wet lab protocols developed by the Tree of Life laboratory are publicly available on protocols.io: https://dx.doi.org/10.17504/protocols.io.8epv5xxy6g1b/v1.

### Sequencing

Pacific Biosciences HiFi circular consensus DNA sequencing libraries were constructed according to the manufacturers’ instructions. Poly(A) RNA-Seq libraries were constructed using the NEB Ultra II RNA Library Prep kit. DNA and RNA sequencing was performed by the Scientific Operations core at the WSI on Pacific Biosciences SEQUEL II (HiFi) and Illumina NovaSeq 6000 (RNA-Seq) instruments. Hi-C data were also generated from tissue of kaClaLepa3 using the Arima2 kit and sequenced on the Illumina NovaSeq 6000 instrument.

### Genome assembly, curation and evaluation

Assembly was carried out with Hifiasm ( Cheng *et al.*, 2021) and haplotypic duplication was identified and removed with purge_dups ( Guan *et al.*, 2020). The assembly was then scaffolded with Hi-C data ( Rao *et al.*, 2014) using YaHS ( Zhou *et al.*, 2023). The assembly was checked for contamination and corrected using the gEVAL system ( Chow *et al.*, 2016) as described previously ( Howe *et al.*, 2021). Manual curation was performed using gEVAL, HiGlass ( Kerpedjiev *et al.*, 2018) and Pretext ( Harry, 2022). The mitochondrial genome was assembled using MitoHiFi ( Uliano-Silva *et al.*, 2023), which runs MitoFinder ( Allio *et al.*, 2020) or MITOS ( Bernt *et al.*, 2013) and uses these annotations to select the final mitochondrial contig and to ensure the general quality of the sequence.

A Hi-C map for the final assembly was produced using bwa-mem2 ( Vasimuddin *et al.*, 2019) in the Cooler file format ( Abdennur & Mirny, 2020). To assess the assembly metrics, the *k*-mer completeness and QV consensus quality values were calculated in Merqury ( Rhie *et al.*, 2020). This work was done using Nextflow ( Di Tommaso *et al.*, 2017) DSL2 pipelines “sanger-tol/readmapping” ( Surana *et al.*, 2023a) and “sanger-tol/genomenote” ( Surana *et al.*, 2023b). The genome was analysed within the BlobToolKit environment ( Challis *et al.*, 2020) and BUSCO scores ( Manni *et al.*, 2021; Simão *et al.*, 2015) were calculated.

Table 3 contains a list of relevant software tool versions and sources.

**Table 3.** Software tools: versions and sources.

Software tool	Version	Source
BlobToolKit	4.1.7	https://github.com/blobtoolkit/blobtoolkit
BUSCO	5.3.2	https://gitlab.com/ezlab/busco
gEVAL	N/A	https://geval.org.uk/
Hifiasm	0.16.1-r375	https://github.com/chhylp123/hifiasm
HiGlass	1.11.6	https://github.com/higlass/higlass
Merqury	MerquryFK	https://github.com/thegenemyers/MERQURY.FK
MitoHiFi	2	https://github.com/marcelauliano/MitoHiFi
PretextView	0.2	https://github.com/wtsi-hpag/PretextView
purge_dups	1.2.3	https://github.com/dfguan/purge_dups
sanger-tol/genomenote	v1.0	https://github.com/sanger-tol/genomenote
sanger-tol/readmapping	1.1.0	https://github.com/sanger-tol/readmapping/tree/1.1.0
YaHS	1.1a.2	https://github.com/c-zhou/yahs

### Wellcome Sanger Institute – Legal and Governance

The materials that have contributed to this genome note have been supplied by a Darwin Tree of Life Partner. The submission of materials by a Darwin Tree of Life Partner is subject to the **‘Darwin Tree of Life Project Sampling Code of Practice’**, which can be found in full on the Darwin Tree of Life website here. By agreeing with and signing up to the Sampling Code of Practice, the Darwin Tree of Life Partner agrees they will meet the legal and ethical requirements and standards set out within this document in respect of all samples acquired for, and supplied to, the Darwin Tree of Life Project.

Further, the Wellcome Sanger Institute employs a process whereby due diligence is carried out proportionate to the nature of the materials themselves, and the circumstances under which they have been/are to be collected and provided for use. The purpose of this is to address and mitigate any potential legal and/or ethical implications of receipt and use of the materials as part of the research project, and to ensure that in doing so we align with best practice wherever possible. The overarching areas of consideration are:

•   Ethical review of provenance and sourcing of the material

•   Legality of collection, transfer and use (national and international)

Each transfer of samples is further undertaken according to a Research Collaboration Agreement or Material Transfer Agreement entered into by the Darwin Tree of Life Partner, Genome Research Limited (operating as the Wellcome Sanger Institute), and in some circumstances other Darwin Tree of Life collaborators.

## Data Availability

European Nucleotide Archive: *Clavelina lepadiformis* (light-bulb sea squirt). Accession number PRJEB57668; https://identifiers.org/ena.embl/PRJEB57668 ( Wellcome Sanger Institute, 2022). The genome sequence is released openly for reuse. The *Clavelina lepadiformis* genome sequencing initiative is part of the Darwin Tree of Life (DToL) project. All raw sequence data and the assembly have been deposited in INSDC databases. The genome will be annotated using available RNA-Seq data and presented through the Ensembl pipeline at the European Bioinformatics Institute. Raw data and assembly accession identifiers are reported in Table 1.

## Author information

Members of the Marine Biological Association Genome Acquisition Lab are listed here: https://doi.org/10.5281/zenodo.8382513.

Members of the Darwin Tree of Life Barcoding collective are listed here: https://doi.org/10.5281/zenodo.4893703.

Members of the Wellcome Sanger Institute Tree of Life Management, Samples and Laboratory Team are listed here: https://doi.org/10.5281/zenodo.10066175.

Members of Wellcome Sanger Institute Scientific Operations: Sequencing Operations are listed here: https://doi.org/10.5281/zenodo.10043364.

Members of the Wellcome Sanger Institute Tree of Life Core Informatics team are listed here: https://doi.org/10.5281/zenodo.10066637.

Members of the Tree of Life Core Informatics collective are listed here: https://doi.org/10.5281/zenodo.5013541.

Members of the Darwin Tree of Life Consortium are listed here: https://doi.org/10.5281/zenodo.4783558.
