# Supplementary material for: The genome sequence of the light-bulb sea squirt, Clavelina lepadiformis (Müller, 1776)
Source: Wellcome Open Res. 2023 Nov 23;8:543. [Version 1] doi: 10.12688/wellcomeopenres.20417.1 (PMC10884593; doi:10.12688/wellcomeopenres.20417.1)
Supplement: Peer review history

# Reviewer report: Reviewer response for version 1

**DOI:** 10.21956/wellcomeopenres.22606.r71066

**Published:** 2024-02-28

**Authors:** Patrick Lemaire

**Affiliations:** 1 Université de Montpellier, Montpellier, France

This report was co-written by Patrick Lemaire and Christelle Dantec (Orcid 0000-0001-7247-6460).

 The reported assembly appears to be of high quality and the assembly and analysis methods used are adequate. This article reports the first high-quality genome of an aplousobranch ascidian. It is thus an important milestone.

 The authors are encouraged to indicate in the methods section the parameters used when running the various pieces of assembly and analysis software. They should also cite previous mitochondrial genome sequencing efforts [1],[2].

 Our main concern relates to the level of confidence that can be given to the species assignment of the sequenced individuals. A photograph is not sufficient, and the authors should describe in greater detail how they convinced themselves that they are reporting a *Clavelina lepadiformis* genome. There is at least one case of innacurate assignment of a tunicate genome to a species [3], a study to which J. Bishop participated. Comparison of the mitochondrial genome of the sequenced individual to two independently sequenced *C. lepadiformis* mitochondrial genomes (see above) could strengthen the authors’ claim.

 The authors should also improve the quality of the text and legends of the article. In the third paragraph of the « Background » section, C. *intestinalis* should be replaced with *C. lepadiformis* ! Also, the legends of figures 3 and 4 (which could be compacted into a single figure) are not intelligible. What does ERR10501016_cov refer to in figure 3? What is the message of this figure? Is there indeed a scaffold with significantly higher GC content than the rest of the assembly? What is it? Likewise, what does « cumulative count » refer to in Figure 4?

Are sufficient details of methods and materials provided to allow replication by others?

Partly

Is the rationale for creating the dataset(s) clearly described?

Yes

Are the datasets clearly presented in a useable and accessible format?

Partly

Are the protocols appropriate and is the work technically sound?

Yes

Reviewer Expertise:

Tunicate biology; Aniseed database

I confirm that I have read this submission and believe that I have an appropriate level of expertise to confirm that it is of an acceptable scientific standard, however I have significant reservations, as outlined above.

## References

[1] : Hypervariability of Ascidian Mitochondrial Gene Order: Exposing the Myth of Deuterostome Organelle Genome Stability. *Molecular Biology and Evolution* .2010;27(2) : 10.1093/molbev/msp234 211-215 10.1093/molbev/msp234 19797354

[2] : Erosion of phylogenetic signal in tunicate mitochondrial genomes on different levels of analysis. *Mol Phylogenet Evol* .2010;55(3) : 10.1016/j.ympev.2010.03.011 860-70 10.1016/j.ympev.2010.03.011 20302953

[3] : Cryptic Diversity and Database Errors Challenge Non-indigenous Species Surveys: An Illustration With Botrylloides spp. in the English Channel and Mediterranean Sea. *Frontiers in Marine Science* .2019;6: 10.3389/fmars.2019.00615 10.3389/fmars.2019.00615

# Reviewer report: Reviewer response for version 1

**DOI:** 10.21956/wellcomeopenres.22606.r71068

**Published:** 2024-02-22

**Authors:** Ferdinand Marlétaz

**Affiliations:** 1 Centre for Life’s Origin & Evolution, Department of Ecology, Evolution & Environment, University College London, London, UK

This manuscript reports the sequencing of the genome of a colonial sea squirt. Given the relatively small number of high-quality tunicate genome this is a nice resource. Overall, the manuscript is clearly written, and procedures appropriately explained. I have two main comments: 

- The authors do not comment on the expected chromosome number in comparison with other tunicates and potentially also in line with previous cytogenetic studies of tunicates. Moreover, tunicates appear to exhibit a particular type of chromosomal architecture (type I according to Hoencamp et al. 2021) in which centromeres and telomeres contact each other with relatively limited contacts elsewhere (as seen with the cross pattern in the HiC map) which could be a problem for HiC scaffolding. 
-  I feel as previously commented for other DToL reports, that the procedure for the HiC scaffolding is insufficiently detailed compared to the ones for DNA extraction and sequencing. Which tissue was used, how was it fixed and/or pulverized? Similarly, YAHS is mentioned but not the details of processing (YAHS takes a BAM file, which should be preprocessed using a tool like pair tools). I think it would really be crucial to release some protocols as the DToL did for the DNA extraction and library making.

Are sufficient details of methods and materials provided to allow replication by others?

Partly

Is the rationale for creating the dataset(s) clearly described?

Yes

Are the datasets clearly presented in a useable and accessible format?

Yes

Are the protocols appropriate and is the work technically sound?

Yes

Reviewer Expertise:

metazoan, genomics, evodevo, phylogeny

I confirm that I have read this submission and believe that I have an appropriate level of expertise to confirm that it is of an acceptable scientific standard.

## References

[1] : 3D genomics across the tree of life reveals condensin II as a determinant of architecture type. *Science* .2021;372(6545) : 10.1126/science.abe2218 984-989 10.1126/science.abe2218 34045355 PMC8172041

# Reviewer report: Reviewer response for version 1

**DOI:** 10.21956/wellcomeopenres.22606.r71065

**Published:** 2023-12-16

**Authors:** Maria Nilsson

**Affiliations:** 1 Senckenberg Biodiversity and Climate Research Centre, Frankfurt am Main, Germany

The authors have sequenced the genome of the light-bulb sea squirt *Clavelina lepadiformis* which is a tunicate. The genome has been sequenced with pacbioHiFi and in addition HiC. The genome size is 0.21 Gbp and the BUSCO completeness is 92.6%. The paper reads well and contains all information necessary to reproduce the work.

Are sufficient details of methods and materials provided to allow replication by others?

Yes

Is the rationale for creating the dataset(s) clearly described?

Yes

Are the datasets clearly presented in a useable and accessible format?

Yes

Are the protocols appropriate and is the work technically sound?

Yes

Reviewer Expertise:

genomics, phylogeny, transposable elements

I confirm that I have read this submission and believe that I have an appropriate level of expertise to confirm that it is of an acceptable scientific standard.
